# Cardiotoxic Effects Following CAR-T Cell Therapy: A Literature Review

**DOI:** 10.1007/s11912-024-01634-2

**Published:** 2025-01-21

**Authors:** Tony Joseph, Jimmy Sanchez, Ahmed Abbasi, Lili Zhang, R. Alejandro Sica, Tim Q. Duong

**Affiliations:** 1 Department of Radiology, Albert Einstein College of Medicine and the Montefiore Medical Center, 111 East 210Th Street, Bronx, NY 10461 USA; 2 Department of Chemistry, CUNY Brooklyn College, 2900 Bedford Ave, Brooklyn, NY 11210 USA; 3 Department of Oncology, Albert Einstein College of Medicine and the Montefiore Medical Center, 111 East 210Th Street, Bronx, NY 10461 USA; 4 Department of Medicine, Cardiology Division, Albert Einstein College of Medicine and the Montefiore Medical Center, 111 East 210Th Street, Bronx, NY 10461 USA

**Keywords:** Cardiotoxicity, Cytokine release syndrome (CRS), Echocardiogram, ECG, EKG, Blood biomarkers, Hypotension, Troponin (TNT), Diastolic dysfunction, Atrial arrhythmia, Cardiomyopathy, Cancer immunotherapy

## Abstract

**Purpose of Review:**

This paper reviewed the current literature on incidence, clinical manifestations, and risk factors of Chimeric Antigen Receptor T-cell (CAR-T) cardiotoxicity.

**Recent Findings:**

CAR-T therapy has emerged as a groundbreaking treatment for hematological malignancies since FDA approval in 2017. CAR-T therapy is however associated with a few side effects, among which cardiotoxicity is of significant concern. There were only a few studies on CAR-T cardiotoxicity published to date with limited sample sizes, and their findings were heterogeneous. It was difficult to reach generalizable conclusions.

**Summary:**

CAR-T therapy was associated with significant risks for acute and subacute cardiotoxicity, as measured by echocardiograms, EKG, and blood biomarkers. Patients with cytokine release syndrome (CRS) grade 2 or higher were more likely to exhibit cardiotoxicity. The most prevalent cardiac events included hypotension-requiring inotropic or vasopressor support, tachycardia, heart failure/decompensation, atrial fibrillation, new or worsening cardiomyopathy, arrhythmia, myocarditis, cardiac arrest, and cardiovascular death. The most prevalent echocardiographic changes were systolic dysfunction and diastolic dysfunction, and abnormal echocardiogram findings. There were differences in findings between adult and pediatric patients. The long-term effects beyond a year post treatment remain largely unknown and long-term follow-up studies are warranted.

**Supplementary Information:**

The online version contains supplementary material available at 10.1007/s11912-024-01634-2.

## Introduction

Chimeric Antigen Receptor T-cell (CAR-T) therapy represents a major advancement in cancer treatment, particularly for patients with refractory or relapsed B-cell malignancies. CAR-T cells are engineered first, collecting T-cells from the patient through leukapheresis [1]. Next, the T-cells are genetically modified to express chimeric antigen receptors (CARs) that recognize specific antigens on cancer cells. Then, the modified T-cells are expanded in culture to sufficient numbers. The CAR-T cells are then reinfused into the patient, where they seek out and destroy cancer cells expressing the target antigen. CAR-T therapy has achieved remarkable clinical success [2, 3]. However, this therapy is not without risks, and one of the potentially serious adverse effects is cardiotoxicity. Understanding the incidence, types of cardiotoxicities, risk factors, underlying mechanisms and clinical management of CAR-T-associated cardiotoxicity is crucial for optimizing patient outcomes.

Cardiotoxicity associated with CAR-T therapy can manifest in several forms, including acute coronary syndrome, heart failure, arrhythmias, and pericarditis/myocarditis [4]. The incidence of cardiotoxicity varies across studies [5]. Cardiotoxic events can occur early, within days to weeks after infusion, or later, highlighting the need for long-term monitoring [6]. Several mechanisms have been proposed for CAR-T-induced cardiotoxicity. One primary mechanism is cytokine release syndrome (CRS), a systemic inflammatory response characterized by elevated levels of cytokines such as interleukin (IL)−6, which can lead to depression of myocardial function and increased permeability of microvasculature [7]. CRS is a clinical diagnosis and usually presents with varying degrees of fever, tachycardia, hypotension, hypoxia, nausea, vomiting, etc. CRS grades range from 1 to 4, as defined by the American Society for Transplantation and Cellular Therapy (Supplemental Table 1) [8]. Direct cardiotoxic effects may occur through the interaction of CAR-T cells with cardiac tissue expressing low levels of target antigens [9]. Off-target effects can result from unintended targeting of antigens shared between malignant and healthy cardiac cells [10]. The only risk factor that was consistently noted across literature for developing cardiotoxicity in CAR-T therapy was the severity of CRS [6, 11]. Serial cardiac monitoring is important, and typical measurements include echocardiogram (ECG), echocardiogram (echo), or cardiac blood biomarkers (e.g., troponin and brain natriuretic peptide (BNP)) to assess cardiac function. The current literature on cardiotoxicity remains sparse and studies typically consist of small sample sizes. The incidence of CAR-T cardiotoxicity, its clinical manifestations and the risk factors for developing CAR-T cardiotoxicity in the literature are highly variable and it is difficult to generalize.

The goal of this paper was to review the literature and characterize the incidence, clinical manifestations, and risk factors associated with CAR-T cardiotoxicity.

## Methods

### Search Strategy and Eligibility Criteria

No ethics committee approval was required for this systematic review. PRIMSA guidelines were used to conduct our systematic review. A literature search was performed from 2017 to June 1st, 2024 using the following search terms that were found in either the title or abstract of the paper: “Chimeric Antigen Receptor T Cell therapy”, “CAR T”, “CAR-T”, or “Chimeric antigen receptors redirect T cell”, and “cardiotoxicity”, “cardiovascular”, cardiac” or “MACE”. Such search terms were used to encompass all phrases of CAR-T therapy. The PubMed database was used to conduct the literature search. We noted that papers relevant to our study were not identified through the PubMed search but was later identified and assessed. Articles that were not relating to CAR-T therapy or its adverse events were removed. Only papers that were written in English were included in this study. The initial search and screening were performed by TJ and independently verified by JS. Oncological therapies other than CAR-T were not reviewed in this study.

## Results and Discussion

**Fig. 1:**
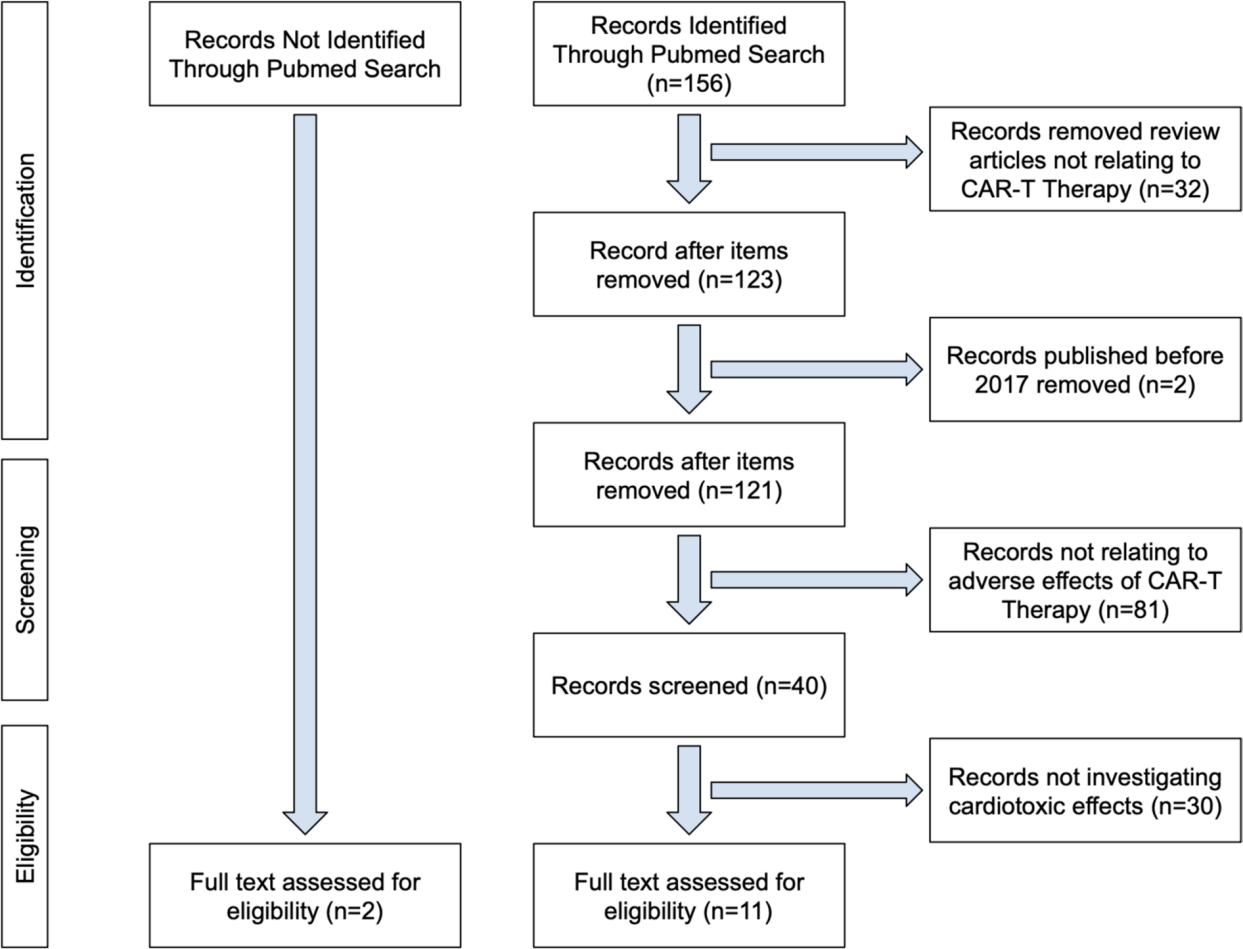
PRIMSA Selection Flowchart

The PubMed search yielded 156 articles, of which 32 were review or perspective articles, 2 were published before 2017, 81 did not report adverse effects of CAR-T therapy, and 30 did not investigate cardiotoxic effects of CAR-T therapy (Fig. 1). Eleven articles were found in the PubMed search after exclusion. In addition, 2 articles were found outside of the PubMed search that discussed cardiotoxic effects of CAR-T therapy.

A total of 13 articles were included in our study. In this review, we described and discussed individual papers in alphabetic orders, followed by summary findings, and future perspectives.

### Summary of Individual Papers

Alvi et al. [12] performed a retrospective cohort study composed of 137 patients, with a median age of 62 (IQR: 54–70). 54 patients developed CRS grade 2 or higher, while 6 patients developed CRS grade 3 or higher. 17 adverse cardiac effects were noted, which included 6 cardiovascular deaths, 6 decompensated HF, and 5 new-onset arrhythmias. All 17 patients who experienced adverse cardiac events had CRS grade 2 or higher. All 6 patients who experienced cardiovascular death has BNP levels greater than 3,000 pg/mL. Of the 29 patients who had pre and post echocardiographic data, 8 patients experienced a reduction in left ventricular ejection fraction (LVEF). Additionally, 53 patients also have pre and post troponin levels taken, of which 29 had an increase in troponin levels after CAR-T infusion. It was noted that patients with increased troponin levels were older (64 ± 9.5 years) in comparison to patients who did not experience elevated troponin levels (58 ± 8.7 years) (p = 0.02). Additionally, certain cardiac risk factors were higher in the elevated troponin cohort, such as diabetes, hypertension, hyperlipidemia, atrial fibrillation/flutter, and CAD, although insignificant (p > 0.05). 16 adverse cardiac events occurred in the elevated troponin cohort, compared to one adverse cardiac event in patients who did not experience elevated troponin. Lastly, CRS grade 2 or higher was associated with elevated troponin levels (p < 0.001) as 83% of the elevated troponin cohort experienced CRS grade 2 or higher, compared to only 33% in the non-elevated troponin cohort.

Brammer et al. [13] performed a retrospective study composed of 90 patients, with an average age of 61 ± 10.9 years. Eighty patients developed CRS, of which 44 patients developed CRS grade 2 or higher. Common symptoms of patients with CRS included hypotension (79 patients) and fever. 17 adverse cardiac events were reported, including 11 patients with arrhythmia, 2 patients with myocarditis, 1 patient with heart failure, and no cardiovascular deaths.

Burstein et al. [14] performed a retrospective study composed of 98 patients, with a median age of 10 (Range: 2–27). 24 patients experienced hypotension-requiring inotropic support. In this cohort, no cardiovascular deaths were observed, how one patient suffered from a cardiac arrest that occurred 2 months after CAR-T infusion. This study noted how of the 24 patients that experienced hypotension-requiring inotropic support, 10 patients presented with abnormal echocardiograms, all of which revealed systolic dysfunction. 13 patients had serum cardiac biomarker data available, of which 12 patients presented with abnormal BNP levels. Evaluation of risk factors for patients with hypotension-requiring inotropic support revealed that no significant differences in age, sex, and race were found between patient cohorts who did or did not experience hypotension-requiring inotropic support. However, patients with baseline characteristics of reduced LVEF (p = 0.019) or diastolic dysfunction (p = 0.021) were observed to have increased rates of hypotension-requiring inotropic support.

Fitzgerald et al. [15] performed a retrospective cohort study composed of 39 patients, with a median age of 11 (Range: 5–22) In total 36 patients developed CRS, with 34 patients developing CRS grade 2 or higher. Common symptoms of CRS included prolonged high fevers, tachycardia, and myalgias. In total, 14 patients had an adverse cardiac event; 13 patients had had profound fluid-refractory vasodilatory shock and 1 patient had cardiomyopathy. This singular patient was also observed to have decreased LVEF values and systolic dysfunction. Organ dysfunction was also noted in 25 patients, which was most commonly hepatic dysfunction. In addition, respiratory failures were also studied. Of the 39 cohort, 8 patients had respiratory failure; of which, 5 patients were later diagnosed with pediatric acute respiratory distress syndrome (PARDS).

Ganatra et al. [16] performed a retrospective cohort study composed of 187 patients, with a median age of 63 (Range: 19–80). 12 patients developed new or worsening cardiomyopathy and experienced a decrease in median LVEF from 58 to 37% after CAR-T infusion. Of these 12 patients, 6 experienced heart failure. 13 patients developed new or worsening arrhythmia, and 86 patients developed CRS grade 2 or higher. 4 patients died in the hospital.

Korell et al. [17] performed a prospective cohort study with 137 patients, with a median age of 60 (Range: 20–83). 25 patients experienced hypotension, 5 patients developed new atrial fibrillation. 75 patients developed CRS, of which 5 patients developed CRS grade 3 or higher. 17 patients developed cardiac decompensation. In terms of echocardiographic findings, 8 patients had abnormal echocardiogram changes, 5 of which were related to atrial fibrillation and 3 of which were unspecific. Additionally, 4 patients had a drop in LVEF measures after CAR-T infusion, of which one patient experienced a decrease of LVEF greater than 10%, while three patients experienced a decrease of LVEF below 50%. Both CRS grade 3 (p < 0.001) or higher and ICANS (p = 0.012) were identified as a risk factor for survival.

Lee et al. [18] performed an observational study on 90 patients, with a median age of 68. A total of 11 adverse cardiac events were reported, 10 patients developed atrial fibrillation, while 1 patient developed cardiomyopathy, which was later reported as acute myocarditis evidenced on cardiac MRI. This patient also presented with abnormal echocardiogram findings and reduced LVEF, which was defined as either a decrease of LVEF greater than 10% or a decrease of LVEF below 50%. Cardiac comorbidities were not found to be significantly different between cardiac events and non-cardiac events groups. Patients in the cardiac event group were statistically older, had higher baseline creatinine, and a larger indexed left atrium volume. 31 patients developed CRS grade 2 or above, of which 6 belonged to the cardiac events group and 25 belonged to the non-cardiac events group. Survival outcomes between the cardiac events group and no-cardiac events group was not significantly different. Of the 88 patients with cardiac biomarker data, there was no difference in Day 5 troponin levels between the cardiac events group and non-cardiac events group. However, all patients in the cardiac events group had BNP levels (125.0 ng/dL) larger (p = 0.019) than the no-cardiac events group (63.0 ng/dL). In this study, abnormal troponin levels were defined as greater than 0.03 ng/mL, and abnormal BNP levels were defined as greater than 100 pg/mL.

Lefebvre et al. (2020) [19] performed a retrospective study composed of 145 patients, with a median age of 60 (IQR: 50–66). 31 patients experienced adverse cardiac events. 2 patients died due to cardiac causes. 21 patients experienced heart failure and 11 patients experienced atrial fibrillation. 2 events of other arrhythmias (supraventricular tachycardia and non-sustained ventricular tachycardia) and 2 events of ACS was noted as well. 104 patients experienced CRS grade 2 or higher. It was also noted that CRS grade 3 and 4 were independently associated with adverse cardiac events. Lastly, it was noted that 22 patients had grade 1 diastolic dysfunction, 1 patient had grade 2 diastolic dysfunction, 3 patients had grade 2 diastolic dysfunction, and 21 patients had indeterminate diastolic function.

Lefebvre et al. (2023) [20] performed a prospective observational study composed of 44 patients, with an average age of 58 (± 11). 11 patients experienced CRS grade 2 or higher. No significant differences in clinical or echocardiographic baseline characteristics were noted in patients who did or did not develop CRS. No significant differences in LVEF were noted as well. In the 23 patients who developed CRS of any grade, 14 patients had elevated BNP levels compared to their baseline characteristics (362 pg/mL vs 63 pg/mL). One patient developed HF and one patient developed atrial fibrillation.

Maude et al. [3] performed a single-center phase 1–2a study composed of 75 patients, with a median age of 11 (Range: 3–23). It was noted that 71 patients had a minimum of one adverse event believed to be due to CAR-T infusion. The most common symptoms of patients after infusion included CRS, pyrexia, decreased appetite, febrile neutropenia, and headache. 35 patients developed CRS grade 3 or higher. Additionally, this study recorded 30 patients experiencing neurologic events, which included encephalopathy, confusional state, delirium, tremor, agitation, somnolence, and seizure. Most of these neurologic events occurred following CRS. Lastly, 13 patients developed hypotension after CAR-T infusion.

Neelapu et al. [21] performed a multicenter, phase 2 trial composed of 101 patients, with a median age of 58 (Range: 23–76). It was noted that all 101 patients experienced an adverse events grade 3 or higher. Of these, the most common effects were neutropenia, anemia, and thrombocytopenia. 53 patients experienced CRS grade 2 or higher, of which the most common symptoms included pyrexia, hypoxia, and hypotension (9 patients). 1 patient with CRS experienced sudden cardiac arrest. No cardiovascular deaths were reported in this study. 39 patients also were diagnosed with tachycardia.

Shalabi et al. [22] performed a retrospective study composed of 52 patients, with a median age of 13 (Range: 4–30). 37 patients developed CRS, of which 23 patients developed CRS grade 2 or higher. Of patients who developed CRS, 6 patients also developed cardiac dysfunction. 36 patients also developed tachycardia, and 9 patients developed hypotension—requiring vasopressor support. In addition, one patient suffered from cardiac arrest. Between the cardiac event group and no cardiac event group, there was no significant difference in baseline LVEF values (p = 0.59). However, of the 13 patients with echocardiographic data, 4 patients experienced both decreased LVEF values and increased troponin levels. Abnormal LVEF values were defined as a greater than 10% absolute decrease in LVEF. Lastly, of the 7 patients with BNP data, 6 patients reported with abnormal BNP levels.

Shouval et al. [23] performed a retrospective cohort study with 236 patients, with a median age of 65 (IQR: 16). 23 patients experienced cardiac arrhythmias, of which 17 patients experienced atrial fibrillation. 108 patients developed CRS grade 2 or higher. In terms of echocardiographic findings, 12 patients had echocardiograms taken within 90 days of an arrhythmic event. Of those, 3 patients experienced decreased LVEF, which was defined as a decrease in LVEF of at least 10% from baseline to a value below 53%. In addition, of patients who experienced an arrhythmic event, 2/14 had elevated cardiac troponin levels and 6/14 had elevated BNP levels.

### Summary findings

We organized summary findings as reports on cardiac events (mostly from ECG), echocardiograms, cardiac blood biomarkers associated with CAR-T cardiotoxicity. We also assessed risk factors and confounding factors associated with CAR-T cardiotoxicity.

### Cardiac Events

Cardiac event summary findings are shown in Table 1. Nine studies [12, 13, 16–21, 23] were in adult (N = 1167) and 4 [3, 14, 15, 22] in pediatric (N = 264) patients, with a combined total of 1,431 patients. Patients had predominately DLBCL, PMBCL, ALL, and MCL cancers. All patients were treated in facilities within the US, except for one study center in Israel [21]. Most studies were performed between 2016 to 2022 but a few were performed in 2010 (CAR-T was approved by the FDA in 2017 [24]). Most follow-up time was less than or about a year post therapy with some reported findings during or soon after CAR-T therapy.

**Table 1:** Summary of cardiac event findings. Age is provided as either mean ± SD, median (IQR), or median (range). Follow up time is provided as a median. All average incidences are averaged with total number of patients as denominator

**Study**	**Location**	**Study Period**	**Follow Up Time**	**Cancer Type**	**Age**	**Race**	**Male/Total**	**Hypotension**^**a**^	**Tachycardia**^**a**^	**Arrhythmia**^**a**^	**CRS** **(grade ≥ 2)**	**HF**	**A-fib**	**CM**	**Myocarditis**	**Cardiac Arrest**	**CV Death**
Alvi 2019	MGH and Moffitt Cancer Center	1/1/16 –11/10/18	10 months	DLBCL:83, TFL:36, MM:11, Other:7	62 (16)	W:126, B:4, O:7	93/137	-	-	5	55	6	3	-	-	3	6
Brammer 2021	Ohio State University’s Cancer Center	1/2016 –12/2019	16.5 months	DLBCL:85, FL:2, MCL:3	61.0 ± 10.9	W:85, O:5	52/90	79^b^	-	11	44	1	-	-	2	-	0
Ganatra 2020	Dana Farber Cancer Institutes, Boston and Ann Arbor	2/2016 –04/2019	5.6 months	DLBCL:137, FL:35, MCL:4, MBL:2, MZL:2, PMBCL:1, others: 6	63 (range:19–80)	W:170, O:17	115/187	-	-	13	86	6	-	12	-	-	4
Korell 2024	University Hospital Heidelberg	10/1/2018 –9/30/2022	3 months	DLBCL:74, PMBCL:3, FL:5, CLL:9, MCL:13, ALL:19, MM:14	60 (16)	-	31/137	25	-	-	5 (CRS ≥ 3)	17	5	-	-	-	-
Lefebvre 2020	Hospital of the U of Pennsylvania	8/2010 –1/2019	15.2 months	ALL:36, DLBCL:43, CLL:66	60 (16)	-	107/145	-	2	2	104	21	11	-	-	-	2
Lefebvre 2023	Hospital of the U of Pennsylvania	6/2019 –2/2022	16.2 months	Lymphoma:43, ALL:1	58 ± 11	W:40, B:3, O:1	34/44	-	-	0	11	1	1	-	-	-	0
Lee 2023	Lee Moffitt Cancer Center	10/2020 –10/2021	10 days	DLBCL:54, TFL:8, B-ALL:3, MCL:25	68 (9.5)	W:86, B:3, O:1	55/90	-	0	0	31	0	10	1	1	-	0
Neelapu 2017	22 study centers (21 in US and 1 in Israel)	11/2015—9/2016	15.4 months	DLBCL:77, PMBCL:8, TFL:16	58 (range:23–76)	-	68/101	9	39	-	53	-	-	-	-	1	0
Shouval 2024	Memorial Sloan Kettering	04/2016–06/2022	30 days	LBCL:215, MCL:14, FL:7	65 (16)	W:191, A:17, B:13, O:15	157/236	-	-	23	108		17	-	-	-	-
**Adult Counts**						W:698/784	712/1167	113	41	54	497	52	47	13	3	4	12
**Adult Average Incidence**						89.0% W	61.0% male	34.5%	12.2%	5.8%	42.6%	6.3%	5.9%	4.7%	1.7%	1.7%	1.5%
Burstein 2018	Children’s Hospital of Philadelphia	4/2012 – 9/2016	6 months	B-ALL:90, T-ALL:1, B lymphoblastic lymp:1, PMBCL:1	10 (range: 2–27)	W:71, B:9, H:9, O:4	51/98	24	-	-	24	-	-	-	-	1	0
Fitzgerald 2017	Children’s Hospital of Philadelphia	4/2012 – 9/2014	up to 28 days	B-ALL:39	11 (range: 5–22)	W:32, B:3, A:2, O:2	20/39	-	-	-	34	-	-	1	-	-	-
Maude 2018	Children’s Hospital of Philadelphia a	4/8/2015 – 4/25/2017	-	ALL:75	11 (range: 3–23)	-	43/75	13	-	-	35 (CRS ≥ 3)	-	-	-	-	-	-
Shalabi 2020	National Cancer Institute	7/2012 – 5/2016	-	ALL:50, NHL:2	13 (range: 4–30)	-	41/52	9	36	0	23	-	-	-	-	1	-
**Ped. Counts**						W:103/137	155/264	46	36	0	116	-	-	1	-	2	0
**Ped. Average Incidence**						75.2% W	58.7% male	20.4%	69.2%	0.0%	43.9%	-	-	2.6%	-	1.3%	0.0%

*Hypotension* Hypotension-requiring inotropic/vasopressor support, *HF* Heart failure / Decompensation, *Cardiomyopathy* New or worsening Cardiomyopathy, *A-Fib* Atrial Fibrillation, *CM* Cardiomyopathy *CV* Cardiovascular, *DLBCL* Diffuse Large B Cell Lymphoma, *TFL* Transformed Follicular Lymphoma, *B-ALL* B-cell Acute Lymphoblastic Leukemia, *LBCL* Large B-cell Lymphoma, *MCL* Mantle Cell Lymphoma, *T-ALL* T-cell Acute Lymphoblastic Leukemia, *PMBCL* Primary Mediastinal Large B-Cell Lymphoma, *MM* Multiple Myeloma, *ALL* Acute Lymphoblastic Leukemia, *CLL* Chronic Lymphocytic Leukemia, *NHL* Non-Hodgkin’s Lymphoma *TFL* Transformed Follicular Lymphoma, *BL*, Burkitt Lymphoma, FL, Follicular Lymphoma *MDL* Mediastinal B-cell Lymphoma, *MZL* Marginal Zone Lymphoma, *W* White, *B* Black, *A* Asian, *O* Other, *H* Hispanic

^a^hypotension, tachycardia, and arrhythmia may occur in the context of CRS, as it is not always described in the papers

^b^includes both Hypotension-requiring and non-requiring inotropic/vasopressor support and patient

Of the adult patients, 60.0% were male and 89.0% were white. Roughly half of all adult patients (42.6%) had CRS grade 2 or higher. The most prevalent cardiotoxic effects were hypotension-requiring inotropic or vasopressor support (34.5%) and tachycardia (12.2%). Note that hypotension and tachycardia are part of the CRS grade definition. The remaining cardiac event findings included heart failure/decompensation (6.3%), atrial fibrillation (5.9%), new or worsening cardiomyopathy (4.7%), arrhythmia (5.8%), myocarditis (1.7%), cardiac arrest (1.7%), and cardiovascular death (1.5%).

Of the pediatric patients, 58.7% were male and 75.2% were white. Pediatric patients had higher incidence of tachycardia (69.2% vs 12.2%) but lower incidence of hypotension (20.4% vs 34.5%) compared to adults. The physiological differences between adult pediatric patients contributed to the differences in tachycardia and hypotension, namely, pediatric patients are more susceptible to tachycardia and less susceptible to hypotension. Pediatric patients showed similar prevalence of CRS grade ≥ 2 (43.9% vs 42.6%), new or worsening cardiomyopathy (2.6% vs 4.7%), arrhythmia (0.0% vs 5.8%), cardiac arrest (1.3% vs 1.7%), and cardiovascular death (0.0% vs 1.5%). Studies on pediatric patients are sparse, with only 4 studies and 264 patients to date.

### Echocardiogram

ECG summary findings are shown in Table 2. Seven studies [12, 16–20, 23] were in adult (N = 976) and 3 [14, 15, 22] in pediatric (N = 189) patients, with a combined total of 1,165 patients. Patients had predominately DLBCL, PMBCL, ALL, and MCL cancers. Most follow-up time was less than or about a year post therapy with some reported findings during or soon after CAR-T therapy.

**Table 2:** Summary of echo findings. Age is provided as either mean ± SD, median (IQR), or median (range). All average incidences are averaged with total number of patients as denominator. White; B:Black;:Asian; O:Other; H:Hispanic

	**Study**	**Location**	**Study Period**	**Follow Up Time**	**Cancer Type**	**Age**	**Male/Total**	**Race**	**CRS (grade ≥ 2)**	**Abnormal Echo**	**Systolic Dysfunction**	**Diastolic Dysfunction**
**Adult**	Alvi 2019	Massachusetts General Hospital and Moffitt Cancer Center	1/1/16—11/10/18	10 months	DLBCL:83, TFL:36, MM:11, Other:7	62 (16)	93/137	W:126, B:4, O:7	55	-	8/29^a^	-
	Ganatra 2020	Dana Farber Cancer Institutes, Boston and Ann Arbor	2/2016—04/2019	5.6 months	DLBCL:137, FL:35, MCL:4, MBL:2, MZL:2, PMBCL:1, others: 6	63 (range:19–80)	115/187	W:170, O:17	86	-	12/116^a^	-
	Korell 2024	University Hospital Heidelberg	10/1/2018—9/30/2022	3 months	DLBCL:74, PMBCL:3, FL:5, CLL:9, MCL:13, ALL:19, MM:14	60 (16)	31/137	-	5 (CRS ≥ 3)	8/137	4/137^a^	-
	Lefebvre 2020	Hospital of the University of Pennsylvania	8/2010—1/2019	15.2 months	ALL:36, DLBCL:43, CLL:66	60 (16)	107/145	-	104	-	-	26/145^b^
	Lefebvre 2023	Hospital of the University of Pennsylvania	6/2019—2/2022	16.2 months	Lymphoma:43, ALL:1	58 ± 11	34/44	W:40, B:3, O:1	11	0/44	0/44^a^	-
	Lee 2023	Lee Moffitt Cancer Center	10/2020—10/2021	10 days	DLBCL:54, TFL:8, B-ALL:3, MCL:25	68 (9.5)	55/90	W:86, B:3, O:1	31	1/90	1/90^a^	-
	Shouval 2024	Memorial Sloan Kettering	04/2016–06/2022	30 days	LBCL:215, MCL:14, FL:7	65 (16)	157/236	W:191, A:17, B:13, O:15	108	-	3/12^e^	-
	**Adult Counts:**						592/976	W:487/557	292	9	25	26
	**Adult Average Incidence:**						60.7% male	87.4% W	47.7%	2.3%	9.0%	17.9%
	Burstein 2018	Children’s Hospital of Philadelphia	4/2012—9/2016	6 months	B-ALL:90, T-ALL:1, B lymphoblastic lymp:1, PMBCL:1	10 (range: 2–27)	51/98	W:71, B:9, H:9, O:4	24	10/24	10/24^c^	-
	Fitzgerald 2017	The Children’s Hospital of Philadelphia	4/2012—9/2014	up to 28 days	B-ALL:39	11 (range: 5–22)	20/39	W:32, B:3, A:2, O:2	34	-	1/39^d^	-
	Shalabi 2020	National Cancer Institute in Bethesda, Maryland	7/2012—5/2016	-	ALL:50, NHL:2	13 (range: 4–30)	41/52	-	23	-	4/13^a^	-
	**Pediatric Counts:**						112/189	W:103/137	81	10	15	-
	**Pediatric Average Incidence:**						59.2% male	75.2% W	42.9%	41.7%	19.7%	-

^a^defined as a reduction was defined as a decrease of at least 10 percentage points in left ventricular ejection fraction (LVEF) to a value below 50%

^b^defined as normal (Grade 0) to impaired relaxation (Grade I), to pseudo normal (Grade II), to restrictive (Grade III), and irreversibly restrictive (Grade IV)

^c^based on echocardiographic evidence from study

^d^not experimentally defined in the study

^e^defined as a decrease in left ventricular ejection fraction (LVEF) of at least 10% from baseline to a value below 53%

Of the adult patients, 60.7% were male and 87.4% were white. Roughly half of all patients (47.7%) had CRS grade 2 or higher. The most prevalent echocardiographic changes were systolic dysfunction (9.0%) and diastolic dysfunction (17.9%). Other echocardiographic changes were categorized as abnormal echocardiogram findings (2.3%). Definitions for abnormal echocardiogram findings varied among papers.

Of the pediatric patients, 59.2% were male and 75.2% were white. Pediatric patients had higher incidence of abnormal echocardiograms (41.7% vs 2.3%) and systolic dysfunction (19.7% vs 8.4%) compared to adults. No pediatric data was available for diastolic dysfunction. Pediatric patients had slightly lower incidences of CRS grade ≥ 2 (42.9% vs 47.7%).

### Blood biomarkers

Biomarker findings are shown in Table 3. There were only 6 [12, 14, 18, 20, 22, 23] studies (N = 657 patients) reporting biomarkers, of which 65.6% were male, and most patients were (85.1%) white. About one-third (38.4%) of all patients had CRS grade ≥ 2. Greater than half (59.5%) of all patients had abnormal BNP levels, and roughly a quarter (26.4%) of patients had abnormal troponin levels. Definitions for abnormal BNP and troponin levels varied significantly across papers.

**Table 3:** Summary of biomarker findings. Age is provided as either mean ± SD, median (IQR), or median (range). All average incidences are averaged with total number of patients as denominator. White; B:Black;:Asian; O:Other; H:Hispanic

Study	Location	Study Period	Follow Up Time	Cancer Type	Age	Race	Male/Total	CRS (grade ≥ 2)	Abnormal Troponin	Abnormal BNP
Alvi 2019	Massachusetts General Hospital and Moffitt Cancer Center	1/1/16—11/10/18	10 months	DLBCL:83, TFL:36, MM:11, Other:7	62 (16)	W:126, B:4, O:7	93/137	55	29/53	-
Burstein 2018	Children’s Hospital of Philadelphia	4/2012—9/2016	6 months	B-ALL:90, T-ALL:1, B lymphoblastic lymp:1, PMBCL:1	10 (range: 2–27)	W:71, B:9, H:9, O:4	51/98	24	-	12/13
Lee 2023	Lee Moffitt Cancer Center	10/2020—10/2021	10 days	DLBCL:54, TFL:8, B-ALL:3, MCL:25	68 (9.5)	W:86, B:3, O:1	55/90	31	0/88	11/88
Lefebvre 2023	Hospital of the University of Pennsylvania	6/2019—2/2022	16.2 months	Lymphoma:43, ALL:1	58 ± 11	W:40, B:3, O:1	34/44	11	-	14/23
Shalabi 2020	National Cancer Institute in Bethesda, Maryland	7/2012—5/2016	-	ALL:50, NHL:2	13 (range: 4–30)	-	41/52	23	4/13	6/7
Shouval 2024	Memorial Sloan Kettering	04/2016–06/2022	30 days	LBCL:215, MCL:14, FL:7	65 (16)	W:191, A:17, B:13, O:15	157/236	108	2/12	6/14
**Counts:**						W:514/605	431/657	252	35	49
**Average Incidence:**						85.1% W	65.6% male	38.4%	26.4%	59.5%

### Risk Factors for Cardiotoxicity

Only four papers statistically modeled risk factors for cardiotoxicity to our knowledge. In the pediatric population, Burstein et al. [14] found pretreatment blast percentage > 25% on bone marrow biopsy was the most predominant factor associated with increased risk for cardiac events (odds ratio, 15.5; 95% CI, 5.1 to 47.1; P < 0.001) (N = 98 patients). Patients with lower ejection fraction (p = 0.019) or diastolic dysfunction (p = 0.021) before treatment had increased rates of hypotension-requiring inotropic support. Notably, pre-existing cardiomyopathy (p = 0.062), TBI (p = 0.629), or higher anthracycline dose (p = 0.444) were not associated with hypotension-requiring inotropic support. In an adult study (n = 236), Shouval et al. [23] found that risk factors for atrial arrhythmia post CAR-T treatment were a history of atrial arrhythmia (OR = 6.80 [2.39–19.6]) and using CAR-T product with a CD28-costimulatory domain (OR = 5.17 [1.72–18.6]) in a multivariable analysis. Lefebvre et al., [19] found risk factors associated with a primary endpoint of MACE (defined as cardiovascular death, symptomatic heart failure, acute coronary syndrome, ischemic stroke and de novo cardiac arrhythmia) included prior atrial fibrillation (*p* = 0.035), aspirin use (*p* = 0.034), statin use (*p* = 0.025), insulin use (*p* = 0.005), baseline creatinine levels (*p* = 0.026), overall CRS grading (*p* < 0.001), CRS grade 2 (*p* = 0.029), CRS grade 3 (*p* = 0.002), CRS grade 4 (*p* < 0.001), diastolic blood pressure (*p* = 0.007), hemoglobin (p = 0.035), platelet count (*p* = 0.027), and a higher mitral E/e’ ratio (*p* = 0.046) using univariable Cox proportional cause-specific hazards regression. Multivariable Cox proportional cause-specific hazards regression analysis determined that baseline creatinine and Grade 3 or 4 CRS were independently associated with MACE. Alvi et al. [12] found a longer duration from the development of CRS to the administration of tocilizumab to be associated with an increased likelihood of having a positive troponin with a hazard ratio analysis (adjusted p = 0.008).

Given the small sample size and the large number of covariates, results from these statistical modeling need to be interpreted with caution.

### Future Perspectives

With CAR-T therapy FDA approval in 2017 [24], it is not surprising that the sample sizes reported in the literature are small. Future studies need to further include larger cohorts, more diverse races and ethnicities to achieve broader generalizability. Future studies should include cardiac computed tomography and magnetic resonance imaging, relevant blood biomarkers, as well as other tests (such as cardiac stress test). Future studies should also include long-term monitoring of cardiac function post CAR-T treatment. The contributions of pre-existing cardiac disease and comorbidities and prior exposure to cardiotoxic therapies to CAR-T cardiotoxicity need further investigation. Statistical modeling is needed to identify risk factors of CAR-T cardiotoxicity. Identifying risk factors could help to refine CAR-T constructs to minimize off-target effects and developing cardioprotective agents for CAR-T therapy.

### Limitations

We did not perform meta-analysis of these cardiotoxicity data from the literature because of small sample sizes and non-uniform reporting. CAR-T also has other side effects (such as neurotoxicity) that were not reviewed in this study.

## Conclusions

CAR-T therapy is associated with significant risks for acute and subacute cardiotoxicity. The long-term effects beyond a year post treatment remain largely unknown and long-term follow-up studies are warranted. Improved understanding of the incidence, clinical manifestation, mechanisms, risk factors, and management strategies for CAR-T-induced cardiotoxicity is essential for maximizing therapeutic efficacy while minimizing adverse cardiotoxicity effects.

## Key References

- Korell, F.; Entenmann, L.; Romann, S.; Giannitsis, E.; Schmitt, A.; Muller-Tidow, C.; Frey, N.; Dreger, P.; Schmitt, M.; Lehmann, L.H. Evaluation of all-cause mortality and cardiovascular safety in patients receiving chimeric antigen receptor T cell therapy: a prospective cohort study. *EClinicalMedicine* **2024**, *69*, 102504, 10.1016/j.eclinm.2024.102504.
  - This prospective cohort study aimed to determine the efficacy of cardiac biomarkers in predicting cardiac events following CAR-T therapy in adult patients, as well as assessing potential cardiotoxicities.
- Lefebvre, B.; Kang, Y.; Vakilpour, A.; Onoue, T.; Frey, N.V.; Brahmbhatt, P.; Huang, B.; Oladuja, K.; Koropeckyj-Cox, D.; Wiredu, C.; et al. Incidence of MACE in Patients Treated With CAR-T Cell Therapy: A Prospective Study. *JACC CardioOncol* **2023**, *5*, 747–754, 10.1016/j.jaccao.2023.07.009.
  - This prospective observational study utilized major adverse cardiovascular events (MACE) as a primary endpoint to study cardiotoxic effects of CAR-T therapy in adult patients.
- Shouval, R.; Goldman, A.; Flynn, J.R.; El-Moghraby, A.; Rehman, M.; Devlin, S.M.; Corona, M.; Landego, I.; Lin, R.J.; Scordo, M.; et al. Atrial arrhythmias following CAR-chimeric antigen receptor T-cell therapy: Incidence, risk factors and biomarker profile. *Br J Haematol* **2024**, 10.1111/bjh.19497.
  - This study analyzes the incidence and risk factors of developing atrial arrhythmias after CAR-T therapy. Inflammatory markers and biomarkers were also analyzed in the post CAR-T therapy setting.

## Supplementary Information

Below is the link to the electronic supplementary material.

Supplementary file1 (DOCX 14 KB)

## Data Availability

No datasets were generated or analysed during the current study.

## References

[1] Zhang C, Liu J, Zhong JF, Zhang X. Engineering CAR-T cells. Biomark Res. 2017;5:22. 10.1186/s40364-017-0102-y. 28652918 10.1186/s40364-017-0102-y PMC5482931

[2] Mitra A, Barua A, Huang L, Ganguly S, Feng Q, He B. From bench to bedside: the history and progress of CAR T cell therapy. Front Immunol. 2023;14:1188049. 10.3389/fimmu.2023.1188049. 37256141 10.3389/fimmu.2023.1188049 PMC10225594

[3] Maude SL, Laetsch TW, Buechner J, Rives S, Boyer M, Bittencourt H, Bader P, Verneris MR, Stefanski HE, Myers GD, et al. Tisagenlecleucel in Children and Young Adults with B-Cell Lymphoblastic Leukemia. N Engl J Med. 2018;378:439–48. 10.1056/NEJMoa1709866. 29385370 10.1056/NEJMoa1709866 PMC5996391

[4] Ghosh AK, Chen DH, Guha A, Mackenzie S, Walker JM, Roddie C. CAR T Cell Therapy-Related Cardiovascular Outcomes and Management: Systemic Disease or Direct Cardiotoxicity? JACC CardioOncol. 2020;2:97–109. 10.1016/j.jaccao.2020.02.011. 34396213 10.1016/j.jaccao.2020.02.011 PMC8352125

[5] Chacko S, Haseeb S, Glover BM, Wallbridge D, Harper A (2018)The role of biomarkers in the diagnosis and risk stratification of acute coronary syndrome. Future science OA. 4(1):FSO251.10.4155/fsoa-2017-0036. 10.4155/fsoa-2017-0036 PMC5729601 29255623

[6] Camilli M, Maggio L, Tinti L, Lamendola P, Lanza GA, Crea F, Lombardo A. Chimeric antigen receptor-T cell therapy-related cardiotoxicity in adults and children cancer patients: A clinical appraisal. Front Cardiovasc Med. 2023;10:1090103. 10.3389/fcvm.2023.1090103. 36895831 10.3389/fcvm.2023.1090103 PMC9988907

[7] Xiao X, Huang S, Chen S, Wang Y, Sun Q, Xu X, Li Y. Mechanisms of cytokine release syndrome and neurotoxicity of CAR T-cell therapy and associated prevention and management strategies. J Exp Clin Cancer Res. 2021;40:367. 10.1186/s13046-021-02148-6. 34794490 10.1186/s13046-021-02148-6 PMC8600921

[8] Lee DW, Santomasso BD, Locke FL, Ghobadi A, Turtle CJ, Brudno JN, Maus MV, Park JH, Mead E, Pavletic S, et al. ASTCT Consensus Grading for Cytokine Release Syndrome and Neurologic Toxicity Associated with Immune Effector Cells. Biol Blood Marrow Transplant. 2019;25:625–38. 10.1016/j.bbmt.2018.12.758. 30592986 10.1016/j.bbmt.2018.12.758 PMC12180426

[9] Rao A, Stewart A, Eljalby M, Ramakrishnan P, Anderson LD Jr, Awan FT, Chandra A, Vallabhaneni S, Zhang K, Zaha VG. Cardiovascular disease and chimeric antigen receptor cellular therapy. Front Cardiovasc Med. 2022;9:932347. 10.3389/fcvm.2022.932347. 36211558 10.3389/fcvm.2022.932347 PMC9538377

[10] Sun S, Hao H, Yang G, Zhang Y, Fu Y. Immunotherapy with CAR-Modified T Cells: Toxicities and Overcoming Strategies. J Immunol Res. 2018;2018:2386187. 10.1155/2018/2386187. 29850622 10.1155/2018/2386187 PMC5932485

[11] Aldoss I, Khaled SK, Budde E, Stein AS. Cytokine Release Syndrome With the Novel Treatments of Acute Lymphoblastic Leukemia: Pathophysiology, Prevention, and Treatment. Curr Oncol Rep. 2019;21:4. 10.1007/s11912-019-0753-y. 30666425 10.1007/s11912-019-0753-y

[12] Alvi RM, Frigault MJ, Fradley MG, Jain MD, Mahmood SS, Awadalla M, Lee DH, Zlotoff DA, Zhang L, Drobni ZD, et al. Cardiovascular Events Among Adults Treated With Chimeric Antigen Receptor T-Cells (CAR-T). J Am Coll Cardiol. 2019;74:3099–108. 10.1016/j.jacc.2019.10.038. 31856966 10.1016/j.jacc.2019.10.038 PMC6938409

[13] Brammer JE, Braunstein Z, Katapadi A, Porter K, Biersmith M, Guha A, Vasu S, Yildiz VO, Smith SA, Buck B, Haddad D. Early toxicity and clinical outcomes after chimeric antigen receptor T-cell (CAR-T) therapy for lymphoma. J ImmunoTher Cancer. 2021;9(8).10.1136/jitc-2020-002303. 10.1136/jitc-2020-002303 PMC8386216 34429331

[14] Burstein DS, Maude S, Grupp S, Griffis H, Rossano J, Lin K. Cardiac Profile of Chimeric Antigen Receptor T Cell Therapy in Children: A Single-Institution Experience. Biol Blood Marrow Transplant. 2018;24:1590–5. 10.1016/j.bbmt.2018.05.014. 29772353 10.1016/j.bbmt.2018.05.014

[15] Fitzgerald JC, Weiss SL, Maude SL, Barrett DM, Lacey SF, Melenhorst JJ, Shaw P, Berg RA, June CH, Porter DL, et al. Cytokine Release Syndrome After Chimeric Antigen Receptor T Cell Therapy for Acute Lymphoblastic Leukemia. Crit Care Med. 2017;45:e124–31. 10.1097/CCM.0000000000002053. 27632680 10.1097/CCM.0000000000002053 PMC5452983

[16] Ganatra S, Redd R, Hayek SS, Parikh R, Azam T, Yanik GA, Spendley L, Nikiforow S, Jacobson C, Nohria A. Chimeric Antigen Receptor T-Cell Therapy-Associated Cardiomyopathy in Patients With Refractory or Relapsed Non-Hodgkin Lymphoma. Circulation. 2020;142:1687–90. 10.1161/CIRCULATIONAHA.120.048100. 33104402 10.1161/CIRCULATIONAHA.120.048100

[17] Korell F, Entenmann L, Romann S, Giannitsis E, Schmitt A, Muller-Tidow C, Frey N, Dreger P, Schmitt M, Lehmann LH. Evaluation of all-cause mortality and cardiovascular safety in patients receiving chimeric antigen receptor T cell therapy: a prospective cohort study. EClinicalMedicine. 2024;69:102504. 10.1016/j.eclinm.2024.102504. 38544797 10.1016/j.eclinm.2024.102504 PMC10965403

[18] Lee DH, Chandrasekhar S, Jain MD, Mhaskar R, Reid K, Lee SB, Corallo S, Hidalgo-Vargas MJ, Kumar A, Chavez J, et al. Cardiac and inflammatory biomarker differences in adverse cardiac events after chimeric antigen receptor T-Cell therapy: an exploratory study. Cardiooncology. 2023;9:18. 10.1186/s40959-023-00170-5. 37005652 10.1186/s40959-023-00170-5 PMC10067156

[19] Lefebvre B, Kang Y, Smith AM, Frey NV, Carver JR, Scherrer-Crosbie M. Cardiovascular Effects of CAR T Cell Therapy: A Retrospective Study. JACC CardioOncol. 2020;2:193–203. 10.1016/j.jaccao.2020.04.012. 32776016 10.1016/j.jaccao.2020.04.012 PMC7413146

[20] Lefebvre B, Kang Y, Vakilpour A, Onoue T, Frey NV, Brahmbhatt P, Huang B, Oladuja K, Koropeckyj-Cox D, Wiredu C, et al. Incidence of MACE in Patients Treated With CAR-T Cell Therapy: A Prospective Study. JACC CardioOncol. 2023;5:747–54. 10.1016/j.jaccao.2023.07.009. 38204993 10.1016/j.jaccao.2023.07.009 PMC10774789

[21] Neelapu SS, Locke FL, Bartlett NL, Lekakis LJ, Miklos DB, Jacobson CA, Braunschweig I, Oluwole OO, Siddiqi T, Lin Y, et al. Axicabtagene Ciloleucel CAR T-Cell Therapy in Refractory Large B-Cell Lymphoma. N Engl J Med. 2017;377:2531–44. 10.1056/NEJMoa1707447. 29226797 10.1056/NEJMoa1707447 PMC5882485

[22] Shalabi H, Sachdev V, Kulshreshtha A, Cohen JW, Yates B, Rosing DR, Sidenko S, Delbrook C, Mackall C, Wiley B, et al. Impact of cytokine release syndrome on cardiac function following CD19 CAR-T cell therapy in children and young adults with hematological malignancies. J Immunother Cancer 2020, 8, 10.1136/jitc-2020-001159 10.1136/jitc-2020-001159 PMC7473612 32883871

[23] Shouval R, Goldman A, Flynn JR, El-Moghraby A, Rehman M, Devlin SM, Corona M, Landego I, Lin RJ, Scordo M, et al. Atrial arrhythmias following CAR-chimeric antigen receptor T-cell therapy: Incidence, risk factors and biomarker profile. Br J Haematol. 2024. 10.1111/bjh.19497. 38735683 10.1111/bjh.19497 PMC11499037

[24] Chen YJ, Abila B, Mostafa Kamel Y. (2023) CAR-T: What Is Next? Cancers (Basel). 2023;15(3):663. 10.3390/cancers15030663. 36765623 10.3390/cancers15030663 PMC9913679

