# Supplementary material for: Cardiotoxic Effects Following CAR-T Cell Therapy: A Literature Review
Source: Curr Oncol Rep. 2025 Jan 21;27(2):135–47. doi: 10.1007/s11912-024-01634-2 (PMC11861112; doi:10.1007/s11912-024-01634-2)
Supplement: Supplementary file 1 — Supplementary file1 (DOCX 14 KB) [file 11912_2024_1634_MOESM1_ESM.docx]

**Table S1.** Grading Scale of Cytokine Release Syndrome (CRS) by the American Society for Transplantation and Cellular Therapy

| **CRS Score** | **Grade 1** | **Grade 2** | **Grade 3** | **Grade 4** |
| --- | --- | --- | --- | --- |
| **Fever** | ≥38°C | ≥38°C | ≥38°C | ≥38°C |
| **Hypotension** | N/A | No vasopressor support | Vasopressor support with or without vasopressin | Multiple vasopressors required (excluding vasopressin) |
| **Hypoxia** | N/A | low-flow nasal cannula or blow-by required | high-flow nasal cannula, facemask, nonrebreather mask, or Venturi mask required | Positive pressure required |
